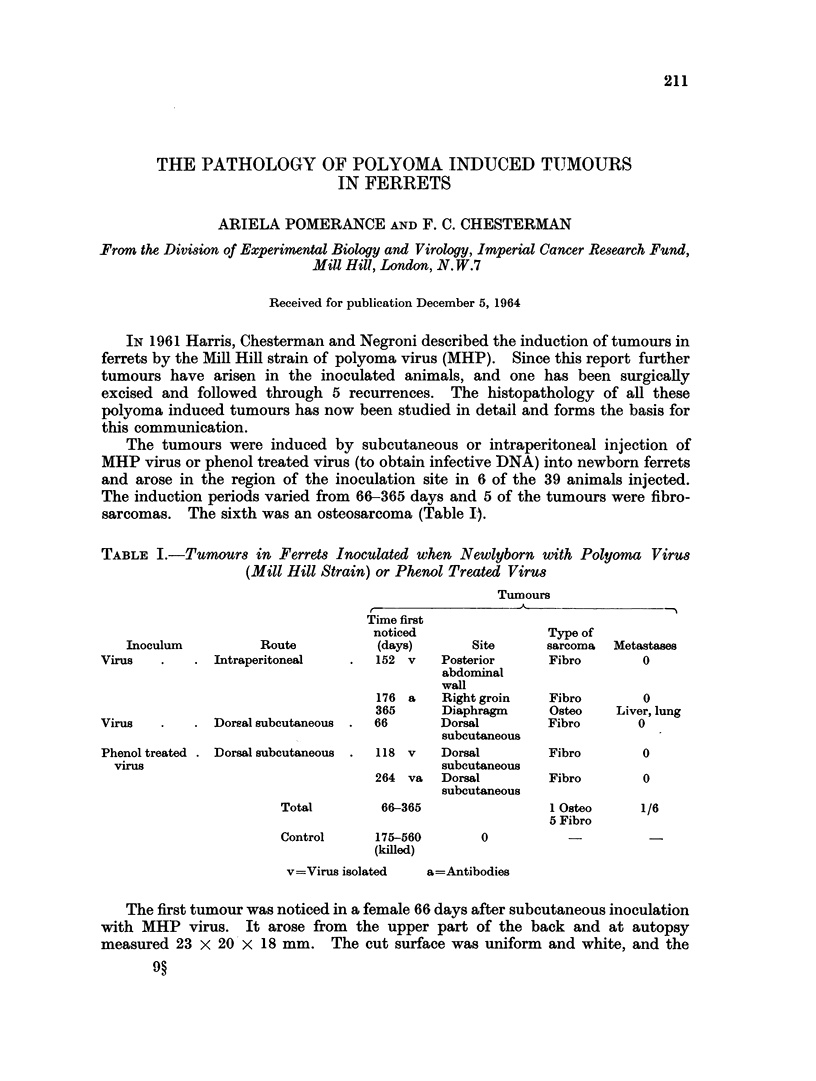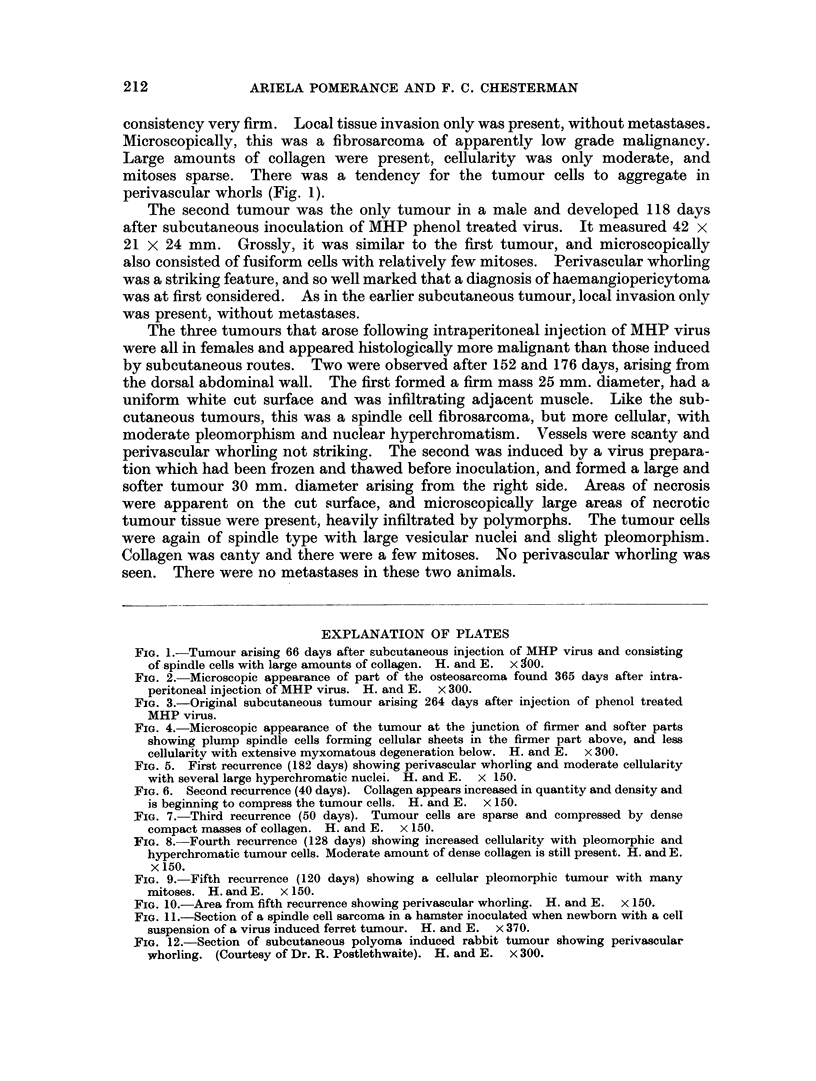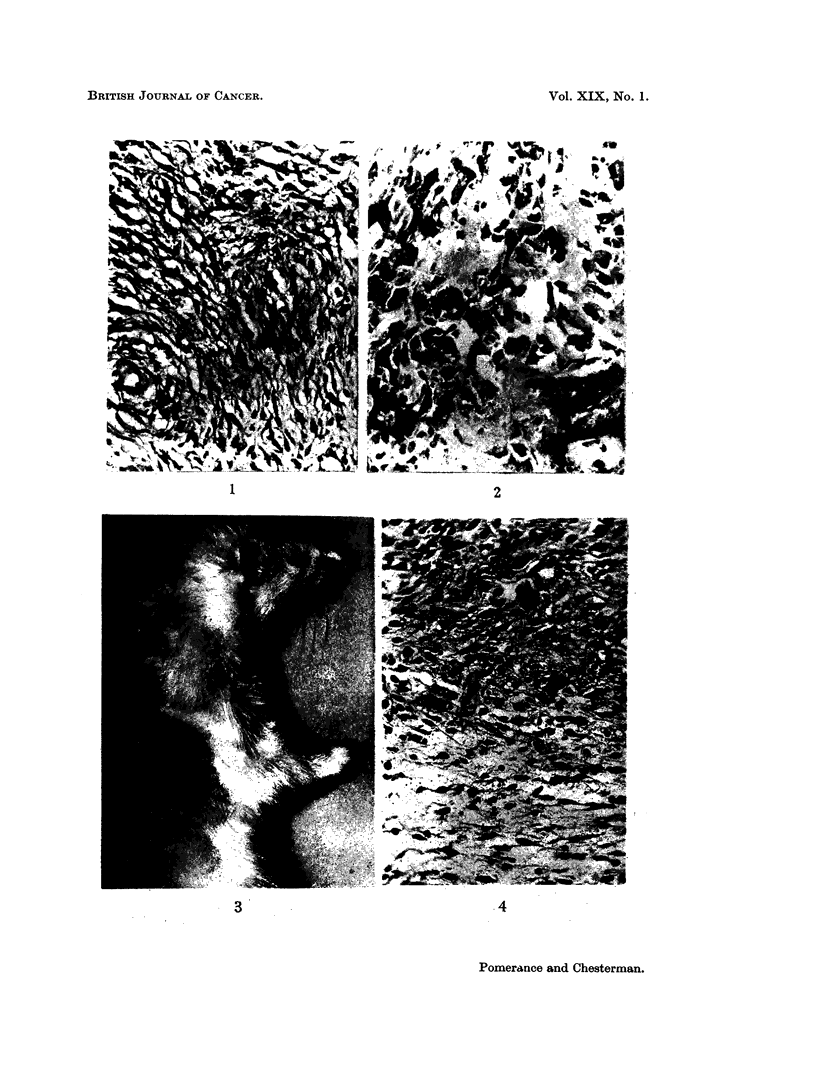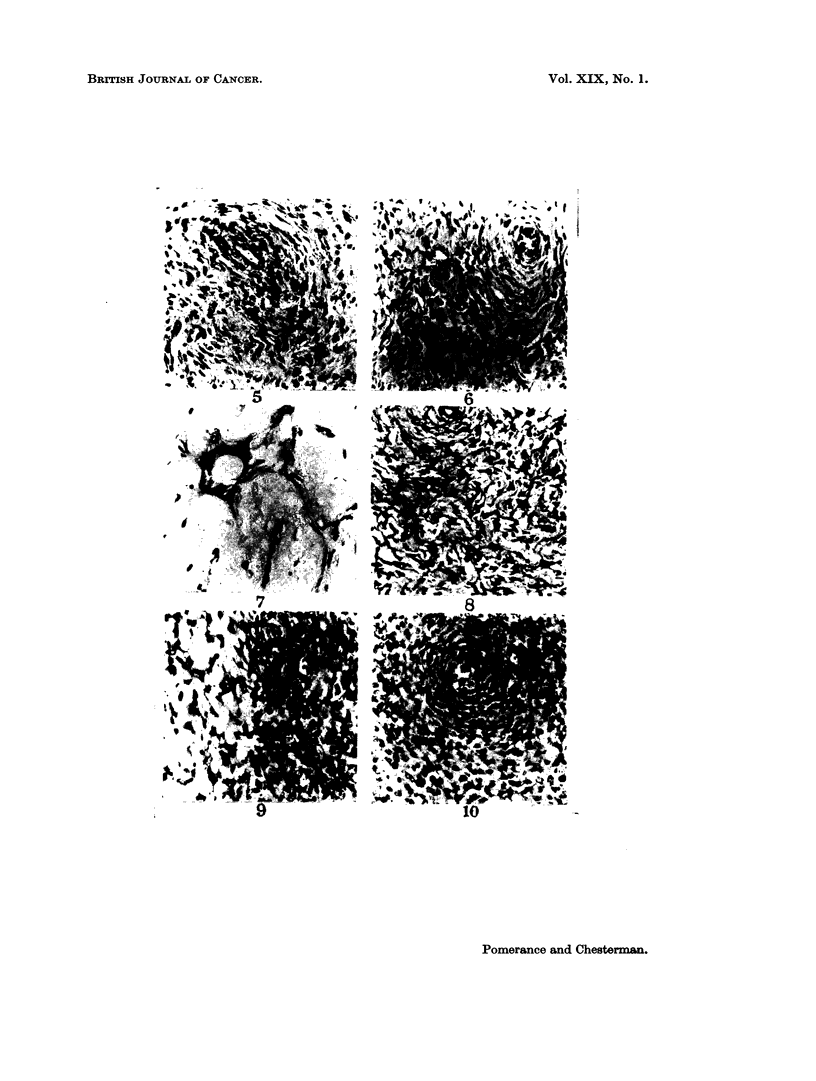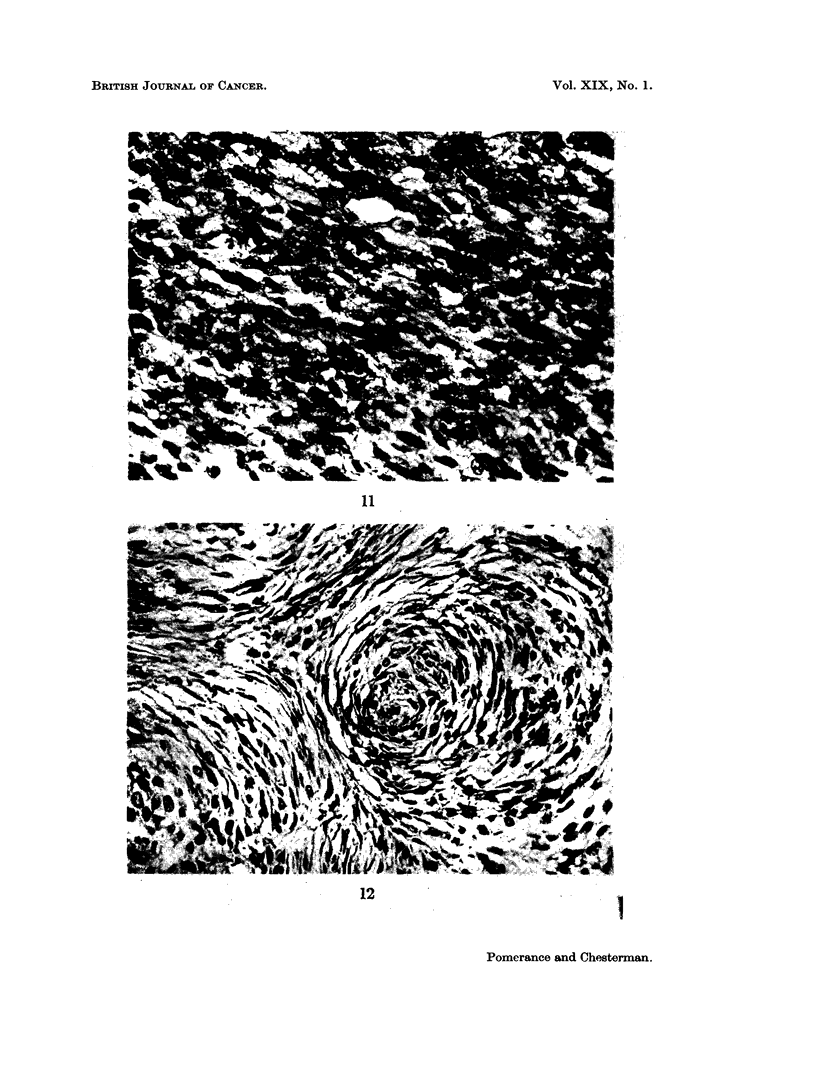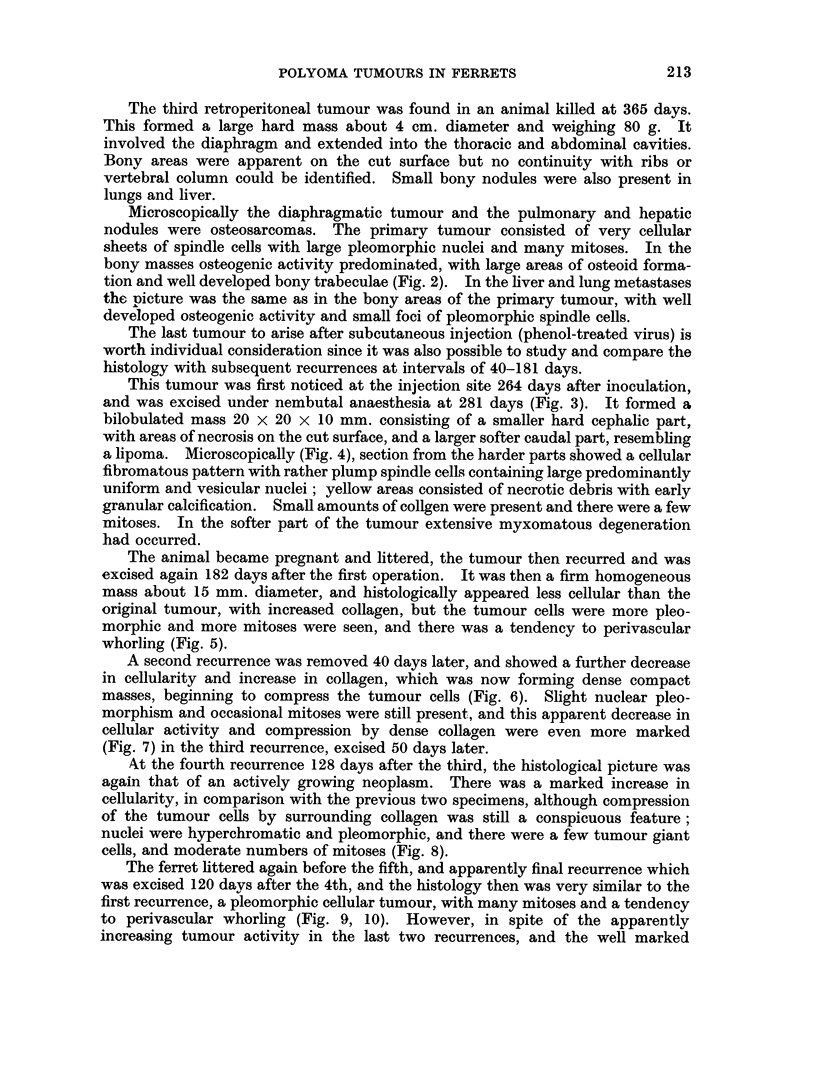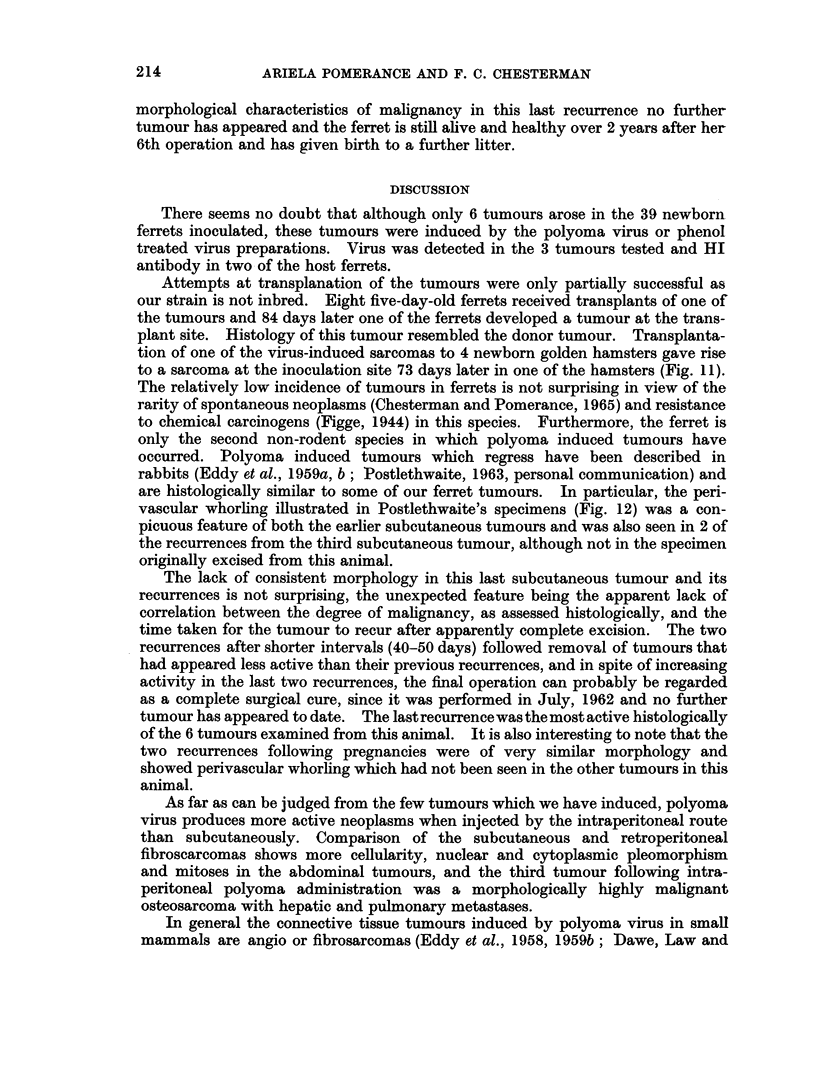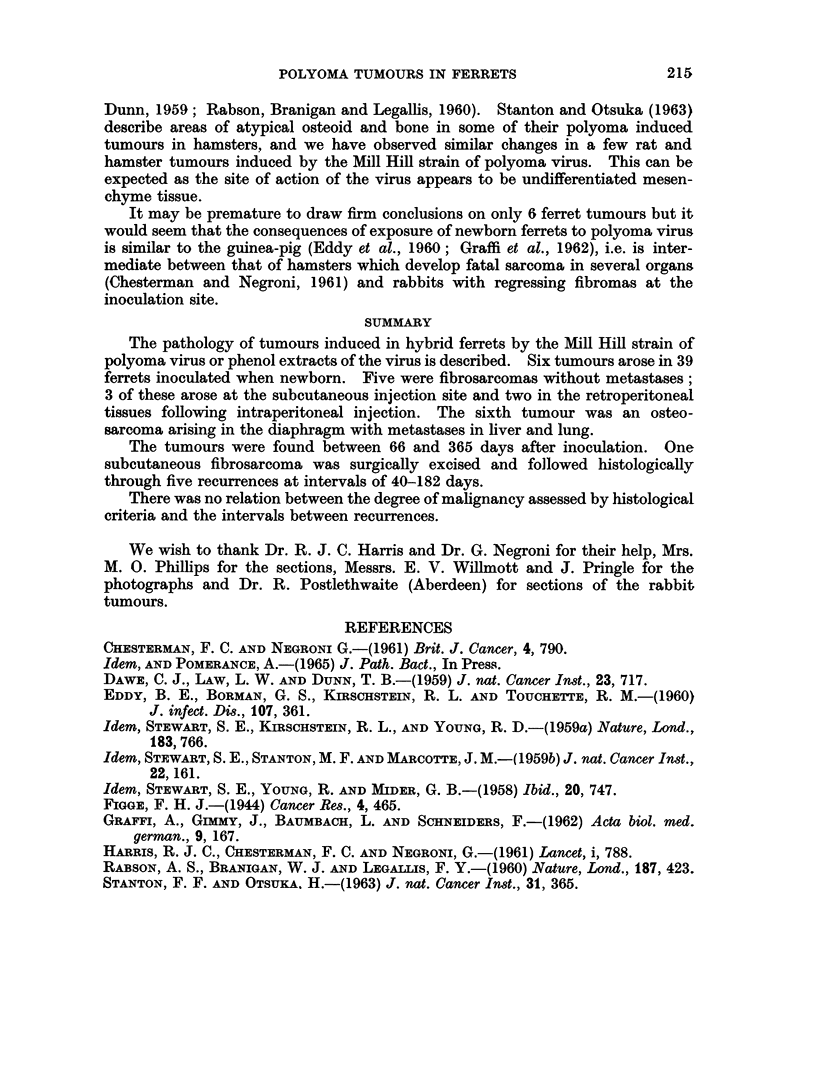# The Pathology of Polyoma Induced Tumours in Ferrets

**DOI:** 10.1038/bjc.1965.26

**Published:** 1965-03

**Authors:** Ariela Pomerance, F. C. Chesterman

## Abstract

**Images:**


### THE PATHOLOGY OF POLYOMA INDUCED TUMOURS IN FERRETS

ARIELA POMERANCE AND F. C. CHESTERMAN

*From the Division of Experimental Biology and Virology, Imperial Cancer Research Fund, Mill Hill, London, N.W.7*

Received for publication December 5, 1964

IN 1961 Harris, Chesterman and Negroni described the induction of tumours in ferrets by the Mill Hill strain of polyoma virus (MHP). Since this report further tumours have arisen in the inoculated animals, and one has been surgically excised and followed through 5 recurrences. The histopathology of all these polyoma induced tumours has now been studied in detail and forms the basis for this communication.

The tumours were induced by subcutaneous or intraperitoneal injection of MHP virus or phenol treated virus (to obtain infective DNA) into newborn ferrets and arose in the region of the inoculation site in 6 of the 39 animals injected. The induction periods varied from 66–365 days and 5 of the tumours were fibrosarcomas. The sixth was an osteosarcoma (Table I).

TABLE I.—*Tumours in Ferrets Inoculated when Newlyborn with Polyoma Virus (Mill Hill Strain) or Phenol Treated Virus*

| | | Tumours | | | |
|---|---|---|---|---|---|
| Inoculum | Route | Time first noticed (days) | Site | Type of sarcoma | Metastases |
| Virus . . | Intraperitoneal . | 152 v | Posterior abdominal wall | Fibro | 0 |
| | | 176 a | Right groin | Fibro | 0 |
| | | 365 | Diaphragm | Osteo | Liver, lung |
| Virus . . | Dorsal subcutaneous . | 66 | Dorsal subcutaneous | Fibro | 0 |
| Phenol treated . virus | Dorsal subcutaneous . | 118 v | Dorsal subcutaneous | Fibro | 0 |
| | | 264 va | Dorsal subcutaneous | Fibro | 0 |
| | Total | 66–365 | | 1 Osteo 5 Fibro | 1/6 |
| | Control | 175–560 (killed) | 0 | — | — |

v=Virus isolated a=Antibodies

The first tumour was noticed in a female 66 days after subcutaneous inoculation with MHP virus. It arose from the upper part of the back and at autopsy measured 23 × 20 × 18 mm. The cut surface was uniform and white, and the consistency very firm. Local tissue invasion only was present, without metastases. Microscopically, this was a fibrosarcoma of apparently low grade malignancy. Large amounts of collagen were present, cellularity was only moderate, and mitoses sparse. There was a tendency for the tumour cells to aggregate in perivascular whorls (Fig. 1).

The second tumour was the only tumour in a male and developed 118 days after subcutaneous inoculation of MHP phenol treated virus. It measured 42 × 21 × 24 mm. Grossly, it was similar to the first tumour, and microscopically also consisted of fusiform cells with relatively few mitoses. Perivascular whorling was a striking feature, and so well marked that a diagnosis of haemangiopericytoma was at first considered. As in the earlier subcutaneous tumour, local invasion only was present, without metastases.

The three tumours that arose following intraperitoneal injection of MHP virus were all in females and appeared histologically more malignant than those induced by subcutaneous routes. Two were observed after 152 and 176 days, arising from the dorsal abdominal wall. The first formed a firm mass 25 mm. diameter, had a uniform white cut surface and was infiltrating adjacent muscle. Like the subcutaneous tumours, this was a spindle cell fibrosarcoma, but more cellular, with moderate pleomorphism and nuclear hyperchromatism. Vessels were scanty and perivascular whorling not striking. The second was induced by a virus preparation which had been frozen and thawed before inoculation, and formed a large and softer tumour 30 mm. diameter arising from the right side. Areas of necrosis were apparent on the cut surface, and microscopically large areas of necrotic tumour tissue were present, heavily infiltrated by polymorphs. The tumour cells were again of spindle type with large vesicular nuclei and slight pleomorphism. Collagen was canty and there were a few mitoses. No perivascular whorling was seen. There were no metastases in these two animals.

---

### EXPLANATION OF PLATES

FIG. 1.—Tumour arising 66 days after subcutaneous injection of MHP virus and consisting of spindle cells with large amounts of collagen. H. and E. ×300.

FIG. 2.—Microscopic appearance of part of the osteosarcoma found 365 days after intraperitoneal injection of MHP virus. H. and E. ×300.

FIG. 3.—Original subcutaneous tumour arising 264 days after injection of phenol treated MHP virus.

FIG. 4.—Microscopic appearance of the tumour at the junction of firmer and softer parts showing plump spindle cells forming cellular sheets in the firmer part above, and less cellularity with extensive myxomatous degeneration below. H. and E. ×300.

FIG. 5. First recurrence (182 days) showing perivascular whorling and moderate cellularity with several large hyperchromatic nuclei. H. and E. × 150.

FIG. 6. Second recurrence (40 days). Collagen appears increased in quantity and density and is beginning to compress the tumour cells. H. and E. ×150.

FIG. 7.—Third recurrence (50 days). Tumour cells are sparse and compressed by dense compact masses of collagen. H. and E. ×150.

FIG. 8.—Fourth recurrence (128 days) showing increased cellularity with pleomorphic and hyperchromatic tumour cells. Moderate amount of dense collagen is still present. H. and E. ×150.

FIG. 9.—Fifth recurrence (120 days) showing a cellular pleomorphic tumour with many mitoses. H. and E. ×150.

FIG. 10.—Area from fifth recurrence showing perivascular whorling. H. and E. ×150.

FIG. 11.—Section of a spindle cell sarcoma in a hamster inoculated when newborn with a cell suspension of a virus induced ferret tumour. H. and E. ×370.

FIG. 12.—Section of subcutaneous polyoma induced rabbit tumour showing perivascular whorling. (Courtesy of Dr. R. Postlethwaite). H. and E. ×300.

1

2

3

4

Pomerance and Chesterman.

11

12

Pomerance and Chesterman.

The third retroperitoneal tumour was found in an animal killed at 365 days. This formed a large hard mass about 4 cm. diameter and weighing 80 g. It involved the diaphragm and extended into the thoracic and abdominal cavities. Bony areas were apparent on the cut surface but no continuity with ribs or vertebral column could be identified. Small bony nodules were also present in lungs and liver.

Microscopically the diaphragmatic tumour and the pulmonary and hepatic nodules were osteosarcomas. The primary tumour consisted of very cellular sheets of spindle cells with large pleomorphic nuclei and many mitoses. In the bony masses osteogenic activity predominated, with large areas of osteoid formation and well developed bony trabeculae (Fig. 2). In the liver and lung metastases the picture was the same as in the bony areas of the primary tumour, with well developed osteogenic activity and small foci of pleomorphic spindle cells.

The last tumour to arise after subcutaneous injection (phenol-treated virus) is worth individual consideration since it was also possible to study and compare the histology with subsequent recurrences at intervals of 40–181 days.

This tumour was first noticed at the injection site 264 days after inoculation, and was excised under nembutal anaesthesia at 281 days (Fig. 3). It formed a bilobulated mass 20 $\times$ 20 $\times$ 10 mm. consisting of a smaller hard cephalic part, with areas of necrosis on the cut surface, and a larger softer caudal part, resembling a lipoma. Microscopically (Fig. 4), section from the harder parts showed a cellular fibromatous pattern with rather plump spindle cells containing large predominantly uniform and vesicular nuclei ; yellow areas consisted of necrotic debris with early granular calcification. Small amounts of collgen were present and there were a few mitoses. In the softer part of the tumour extensive myxomatous degeneration had occurred.

The animal became pregnant and littered, the tumour then recurred and was excised again 182 days after the first operation. It was then a firm homogeneous mass about 15 mm. diameter, and histologically appeared less cellular than the original tumour, with increased collagen, but the tumour cells were more pleomorphic and more mitoses were seen, and there was a tendency to perivascular whorling (Fig. 5).

A second recurrence was removed 40 days later, and showed a further decrease in cellularity and increase in collagen, which was now forming dense compact masses, beginning to compress the tumour cells (Fig. 6). Slight nuclear pleomorphism and occasional mitoses were still present, and this apparent decrease in cellular activity and compression by dense collagen were even more marked (Fig. 7) in the third recurrence, excised 50 days later.

At the fourth recurrence 128 days after the third, the histological picture was again that of an actively growing neoplasm. There was a marked increase in cellularity, in comparison with the previous two specimens, although compression of the tumour cells by surrounding collagen was still a conspicuous feature ; nuclei were hyperchromatic and pleomorphic, and there were a few tumour giant cells, and moderate numbers of mitoses (Fig. 8).

The ferret littered again before the fifth, and apparently final recurrence which was excised 120 days after the 4th, and the histology then was very similar to the first recurrence, a pleomorphic cellular tumour, with many mitoses and a tendency to perivascular whorling (Fig. 9, 10). However, in spite of the apparently increasing tumour activity in the last two recurrences, and the well marked morphological characteristics of malignancy in this last recurrence no further tumour has appeared and the ferret is still alive and healthy over 2 years after her 6th operation and has given birth to a further litter.

### DISCUSSION

There seems no doubt that although only 6 tumours arose in the 39 newborn ferrets inoculated, these tumours were induced by the polyoma virus or phenol treated virus preparations. Virus was detected in the 3 tumours tested and HI antibody in two of the host ferrets.

Attempts at transplanation of the tumours were only partially successful as our strain is not inbred. Eight five-day-old ferrets received transplants of one of the tumours and 84 days later one of the ferrets developed a tumour at the transplant site. Histology of this tumour resembled the donor tumour. Transplantation of one of the virus-induced sarcomas to 4 newborn golden hamsters gave rise to a sarcoma at the inoculation site 73 days later in one of the hamsters (Fig. 11). The relatively low incidence of tumours in ferrets is not surprising in view of the rarity of spontaneous neoplasms (Chesterman and Pomerance, 1965) and resistance to chemical carcinogens (Figge, 1944) in this species. Furthermore, the ferret is only the second non-rodent species in which polyoma induced tumours have occurred. Polyoma induced tumours which regress have been described in rabbits (Eddy *et al.*, 1959*a, b* ; Postlethwaite, 1963, personal communication) and are histologically similar to some of our ferret tumours. In particular, the perivascular whorling illustrated in Postlethwaite's specimens (Fig. 12) was a conpicuous feature of both the earlier subcutaneous tumours and was also seen in 2 of the recurrences from the third subcutaneous tumour, although not in the specimen originally excised from this animal.

The lack of consistent morphology in this last subcutaneous tumour and its recurrences is not surprising, the unexpected feature being the apparent lack of correlation between the degree of malignancy, as assessed histologically, and the time taken for the tumour to recur after apparently complete excision. The two recurrences after shorter intervals (40–50 days) followed removal of tumours that had appeared less active than their previous recurrences, and in spite of increasing activity in the last two recurrences, the final operation can probably be regarded as a complete surgical cure, since it was performed in July, 1962 and no further tumour has appeared to date. The last recurrence was the most active histologically of the 6 tumours examined from this animal. It is also interesting to note that the two recurrences following pregnancies were of very similar morphology and showed perivascular whorling which had not been seen in the other tumours in this animal.

As far as can be judged from the few tumours which we have induced, polyoma virus produces more active neoplasms when injected by the intraperitoneal route than subcutaneously. Comparison of the subcutaneous and retroperitoneal fibroscarcomas shows more cellularity, nuclear and cytoplasmic pleomorphism and mitoses in the abdominal tumours, and the third tumour following intraperitoneal polyoma administration was a morphologically highly malignant osteosarcoma with hepatic and pulmonary metastases.

In general the connective tissue tumours induced by polyoma virus in small mammals are angio or fibrosarcomas (Eddy *et al.*, 1958, 1959*b* ; Dawe, Law and

Dunn, 1959 ; Rabson, Branigan and Legallis, 1960). Stanton and Otsuka (1963) describe areas of atypical osteoid and bone in some of their polyoma induced tumours in hamsters, and we have observed similar changes in a few rat and hamster tumours induced by the Mill Hill strain of polyoma virus. This can be expected as the site of action of the virus appears to be undifferentiated mesenchyme tissue.

It may be premature to draw firm conclusions on only 6 ferret tumours but it would seem that the consequences of exposure of newborn ferrets to polyoma virus is similar to the guinea-pig (Eddy *et al.*, 1960 ; Graffi *et al.*, 1962), i.e. is intermediate between that of hamsters which develop fatal sarcoma in several organs (Chesterman and Negroni, 1961) and rabbits with regressing fibromas at the inoculation site.

SUMMARY

The pathology of tumours induced in hybrid ferrets by the Mill Hill strain of polyoma virus or phenol extracts of the virus is described. Six tumours arose in 39 ferrets inoculated when newborn. Five were fibrosarcomas without metastases ; 3 of these arose at the subcutaneous injection site and two in the retroperitoneal tissues following intraperitoneal injection. The sixth tumour was an osteosarcoma arising in the diaphragm with metastases in liver and lung.

The tumours were found between 66 and 365 days after inoculation. One subcutaneous fibrosarcoma was surgically excised and followed histologically through five recurrences at intervals of 40–182 days.

There was no relation between the degree of malignancy assessed by histological criteria and the intervals between recurrences.

We wish to thank Dr. R. J. C. Harris and Dr. G. Negroni for their help, Mrs. M. O. Phillips for the sections, Messrs. E. V. Willmott and J. Pringle for the photographs and Dr. R. Postlethwaite (Aberdeen) for sections of the rabbit tumours.

### REFERENCES

CHESTERMAN, F. C. AND NEGRONI G.—(1961) *Brit. J. Cancer*, **4**, 790.

*Idem*, AND POMERANCE, A.—(1965) *J. Path. Bact.*, In Press.

DAWE, C. J., LAW, L. W. AND DUNN, T. B.—(1959) *J. nat. Cancer Inst.*, **23**, 717.

EDDY, B. E., BORMAN, G. S., KIRSCHSTEIN, R. L. AND TOUCHETTE, R. M.—(1960) *J. infect. Dis.*, **107**, 361.

*Idem*, STEWART, S. E., KIRSCHSTEIN, R. L., AND YOUNG, R. D.—(1959*a*) *Nature, Lond.*, **183**, 766.

*Idem*, STEWART, S. E., STANTON, M. F. AND MARCOTTE, J. M.—(1959*b*) *J. nat. Cancer Inst.*, **22**, 161.

*Idem*, STEWART, S. E., YOUNG, R. AND MIDER, G. B.—(1958) *Ibid.*, **20**, 747.

FIGGE, F. H. J.—(1944) *Cancer Res.*, **4**, 465.

GRAFFI, A., GIMMY, J., BAUMBACH, L. AND SCHNEIDERS, F.—(1962) *Acta biol. med. german.*, **9**, 167.

HARRIS, R. J. C., CHESTERMAN, F. C. AND NEGRONI, G.—(1961) *Lancet*, i, 788.

RABSON, A. S., BRANIGAN, W. J. AND LEGALLIS, F. Y.—(1960) *Nature, Lond.*, **187**, 423.

STANTON, F. F. AND OTSUKA. H.—(1963) *J. nat. Cancer Inst.*, **31**, 365.